# A Discriminative Long Short Term Memory Network with Metric Learning Applied to Multispectral Time Series Classification

**DOI:** 10.3390/jimaging6070068

**Published:** 2020-07-12

**Authors:** Merve Bozo, Erchan Aptoula, Zehra Çataltepe

**Affiliations:** 1 Department of Computer Engineering, Istanbul Technical University, Maslak, Istanbul 34469, Turkey; cataltepe@itu.edu.tr; 2 Institute of Information Technologies, Gebze Technical University, Kocaeli 41400, Turkey; eaptoula@gtu.edu.tr

**Keywords:** multitemporal, multispectral, long short-term memory network, metric learning

## Abstract

In this article, we propose an end-to-end deep network for the classification of multi-spectral time series and apply them to crop type mapping. Long short-term memory networks (LSTMs) are well established in this regard, thanks to their capacity to capture both long and short term temporal dependencies. Nevertheless, dealing with high intra-class variance and inter-class similarity still remain significant challenges. To address these issues, we propose a straightforward approach where LSTMs are combined with metric learning. The proposed architecture accommodates three distinct branches with shared weights, each containing a LSTM module, that are merged through a triplet loss. It thus not only minimizes classification error, but enforces the sub-networks to produce more discriminative deep features. It is validated via *Breizhcrops*, a very recently introduced and challenging time series dataset for crop type mapping.

## 1. Introduction

The proliferation of satellites in the last decade has resulted in wider public access to remote sensing images with progressively higher spectral, spatial and temporal resolutions. For instance, contrary to the past when multi-spectral images where scarce, thanks to satellites such as Sentinel-2A/B, multi-spectral images of the entire globe are now acquired regularly every few days and are publicly available. Obviously, acquisitions across multiple dates of the same geographical scene, are invaluable for land cover map production, since it enables better distinguishing between thematic classes with spectral responses varying across time. Crop type classification is evidently one of its paramount applications [1]. Nevertheless, the presence of both multiple temporal instances and spectral bands for a given scene constitutes a significant methodological challenge in terms of their compound description [2], and has led to a large number of published work to this end [3].

One way of categorizing the related works can be in terms of whether they rely on feature engineering or automatically produced deep learning features. In the case of the former, machine learning models receive commonly as input directly spectral pixel signatures, various ancillary parameters, simple statistics thereof, as well as spectral indices relevant to the data domain, such as the normalized difference vegetation index [4]. They are commonly combined with various dimension reduction techniques [5]. Long term temporal relations are captured via features designed through the domain knowledge of experts (e.g., amplitude of cropping cycles, etc.) [6]. Temporal distortions constitute an important issue that reduces significantly the generalization potential of handcrafted features. It is often addressed through Dynamic Time Warping [1,7,8,9]. Once the features have been extracted, almost the entire machine learning arsenal has been explored in one way or another for their classification: artificial neural networks [10], clustering algorithms [11,12], decision trees [13] random forests [14,15] and support vector machines [16] to name a few. For a comprehensive review of land cover classification through time series the reader is referred to [17].

In parallel to the aforementioned developments, the world of machine learning/pattern recognition has experienced a fundamental paradigm-shift in the last few years due to deep learning [18]. Ground-breaking performance was achieved in various highly challenging computer vision tasks, especially via convolutional neural networks (CNNs) [19]. This new family of algorithms enabling the design and efficient training of deep neural networks has rendered de facto redundant the process of feature engineering, as it is now delegated to the network itself. In addition, depending on the quality as well as amount of provided training data, deep networks often compute features vastly outperforming handcrafted alternatives. For a survey of deep learning advances in remote sensing cf. [20]. CNNs however specialize in exploiting spatial inter-pixel relations, recurrent neural networks (RNNs) on the other hand represent the branch of deep learning most suitable to tackle temporal data relations. Consequently, they have been intensively studied in the context of multi-temporal remote sensing data analysis. Especially long short-term memory (LSTM) networks [21], a variant of RNNs solving their vanishing gradient problem, have been particularly popular in this context. Their direct applications to multitemporal data has been investigated through a variety of sensors, including Sentinel-1 SAR [22] and Landsat [23]. Soon after, their combinations with CNNs were investigated, both in the mono-dimensional case [24] as well as with 2-dimensional CNNs [25,26,27]. In further studies, LSTMs have been applied to crop classification through Sentinel-2 data, without even radiometric or geometric preprocessing [28,29] and have still achieved state-of-the-art performance. As of lately, new variants of CNNs have also appeared to this end, named *temporal CNNs* that apply convolutions on the temporal domain instead of the spatial domain [30].

High intra-class variance and inter-class similarity is an issue common to all classification problems. In this particular context, it can manifest itself as a thematic class with different spectral/temporal profiles depending on its geographical location. A general way of battling it is metric learning [31], and deep metric learning in particular. It denotes the process of learning a metric from the data, such that samples of the same class are closer to each other while ensuring that the samples from different classes are distanced. As such it can be used to reduce intra-class variance and increase the inter-class variance of the calculated features. Following the aforementioned success and wide use of LSTMs in the field of multi-temporal remote sensing analysis, in this article we also adopt them as our primary tool and propose to combine them with metric learning, in an effort to produce more discriminative features. As a concept, the combined use of metric learning with CNNs has been already reported with promising results for hyperspectral image classification [32] and optical scene recognition [33,34,35].

The contribution of this article is an exploration of the combined use of LSTMs with deep metric learning in the context of remote sensing time series classification. We propose an architecture consisting of three identical LSTMs with shared weights. We employ weighted cross-entropy loss for classification and triplet loss for metric learning. Thus, our network is taught not only to produce features that lead to correct classification, but also features with low intra-class variance and high inter-class variance, hence potentially leading to higher performance. The explored approach is validated through the very recently introduced *Breizhcrops* benchmark dataset of Sentinel-2 agricultural time series, acquired across an entire calendar year.

The next section (Section 2) will detail the investigated method, then Section 3 will present the results of the conducted experiments, and finally Section 4 is devoted to concluding remarks.

## 2. Methodology

This section will start with background information on metric learning, and then continue by elaborating on its combination with LSTMs. Specifically, we will introduce the overall architecture and then explain how the combined loss is calculated.

### 2.1. Background

End-to-end deep metric learning networks have the ability to learn feature embeddings directly from the data and to map them in an embedding space where inter-class distances are more emphasized and intra-class variances are decreased. Overall, they aggregate the feature extraction and similarity comparison stages [36].

**Figure 1:**
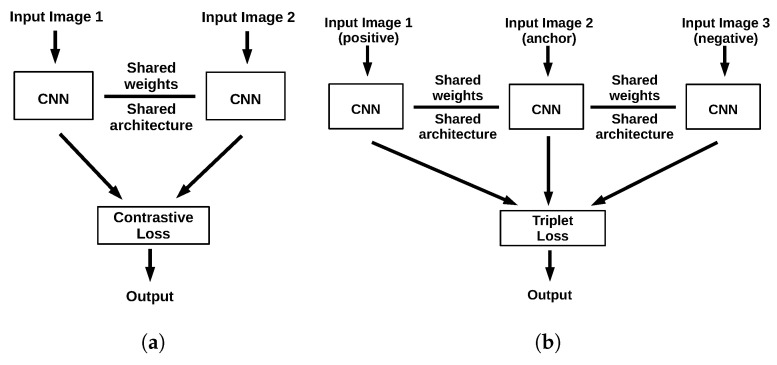
Outlines of common metric learning architectures. (**a**) Siamese architecture. (**b**) Triplet architecture.

The Siamese Network [36] pioneered deep metric learning and has rapidly achieved remarkable results in various recognition tasks [32,34,35,37]. Siamese networks receive as input a pair of samples and compute feature embeddings with their sub-networks possessing the same architecture and shared weights. The loss function of the overall network, often in the form of *contrastive loss* [38] aims to minimize the distance between the feature embeddings of samples of the same class, and increase the distance of samples coming from distinct classes (Figure 1a).

Triplet networks [39] on the other hand, are inspired by Siamese networks and possess three sub-networks. In contrast with Siamese networks, that might be provided either with samples of the same class, or not. triplet networks are always provided with three samples, two of the same class (the *anchor* and the *positive* sample) and one of different class than the anchor (the *negative* sample). The feature embedding of each is computed using its respective sub-network (Figure 1b). The network’s overall loss (*triplet loss* [39]) is computed in such a way, so as to minimize the distance between the embeddings of the anchor and the positive sample, while increasing the distance between the embeddings of the anchor and the negative sample. Triplet networks have demonstrated a consistently superior performance with respect to Siamese networks [39].

### 2.2. A More Discriminative LSTM

Recurrent neural networks, receive as input series of observations, and process them sequentially, producing and updating a feature vector that preserves contextual information. LSTMs on the other hand, which constitute an instance of *gated* RNNs, employ sub-networks (i.e., gates) whose goal is to parameterize this contextual information, so as to overcome the vanishing gradient problem [21].

In the light of the aforementioned (Section 2.1) developments, we have chosen to combine LSTMs with metric learning via the triplet paradigm. Our objective is to reinforce LSTMs’ capacity of exploiting temporal relations, through metric learning. Our hypothesis is that this enables the network to produce more discriminative features by decreasing their intra-class variance and increasing inter-class variance.

**Figure 2:**
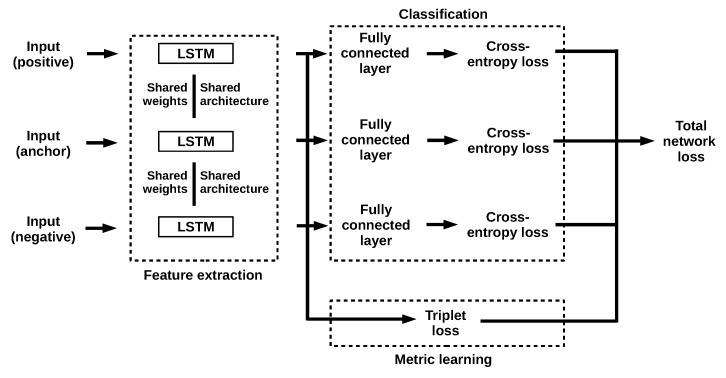
The overall network architecture of the proposed approach.

The proposed *Triplet LSTM Network* consists of three branches (i.e., sub-networks) with shared weights and architecture (Figure 2). The overall network receives as input a time series triplet, $\left(x_{p},x_{a},x_{n}\right)$, where $x_{a}$ denotes the anchor, $x_{p}$ the positive and $x_{n}$ the negative sample. Feature embeddings are generated through the LSTM sub-networks that constitute the branches of the Triplet network. The resulting feature embeddings are provided to the classification and metric learning stages concurrently. The similarity of the embeddings is calculated via triplet loss in the metric learning stage, and classification loss is calculated through weighted cross-entropy. The weighted sum of the calculated losses is then employed as the total network loss and used for back-propagation. Now let us elaborate on each of these steps, starting from feature embedding, and advancing through classification loss, metric loss all the way to the network’s combined loss function.

Given an input sample, an LSTM network produces feature embeddings. Our three LSTMs have each 128 hidden dimensions (the same number as in [40]), and receive as input time series samples in the form of triplets: $\left(x_{p},x_{a},x_{n}\right)$. Each LSTM acts as a feature extractor, calculating a feature embedding of its input through the learned function f(·).

Following the calculation of feature embeddings, a fully connected layer takes place combined with the *softmax* function (Equation (1)) to produce class probabilities:

(1) $$\sigma {\left(f\left(x\right)\right)}^{\left(i\right)}=\frac{e^{f{\left(x\right)}^{\left(i\right)}}}{\sum_{j}^{n}e^{f{\left(x\right)}^{\left(j\right)}}}$$

where $f{\left(x\right)}^{\left(i\right)}$ denotes the *i*-th dimension of the vector f(x). In more detail, softmax $\sigma :R^{n}\to R^{n}$ receives as input the unnormalized vector f(x) produced by the fully connected layer, and outputs once again a *n*-dimensional vector, where *n* is the number of classes, representing the estimated class probability distribution for *x*. Then classification loss is calculated through the commonly employed weighted cross-entropy loss [41]:

(2) $$L_{c}\left(x,y\right)=-\sum_{k}^{n}w_{k}\cdot y^{\left(k\right)}\cdot log\left(\sigma \left(f{\left(x\right)}^{\left(k\right)}\right)\right)$$

where $y={\left(y^{\left(1\right)},\cdots ,y^{\left(n\right)}\right)}^{T}$ is the one-hot vector of labels associated with sample *x*, and $y^{\left(k\right)}$ represents a binary indicator of whether the correct class label is *k*, and $\sigma (f{\left(x\right)}^{\left(k\right)})$ denotes the estimated probability of class *k*. To tackle the imbalance of classes in the training set, and the eventual undesired dominance of over-represented classes during training, we additionally employ the weight factor $w_{k}=\tilde{g}/g\left(k\right)$, with g(k) representing the number of training samples of class *k* divided by the total number of samples in the training set, and $\tilde{g}$ is the median of the *n* class frequencies; this is commonly known as inverse median frequency weighting [42]. It thus penalizes highly classification errors with under-represented classes.

**Figure 3:**
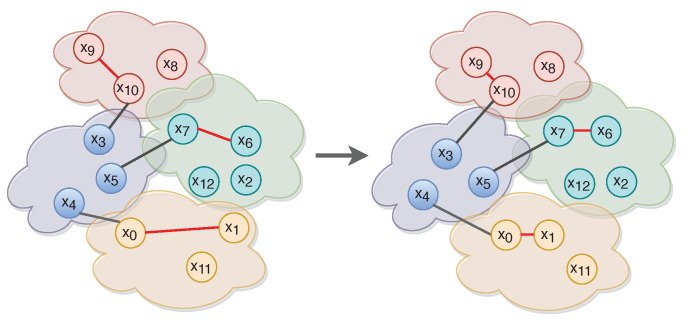
Triplet loss pairs every anchor ($x_{0}$, $x_{7}$, $x_{10}$) with a single positive ($x_{1}$, $x_{6}$, $x_{9}$) and negative ($x_{4}$, $x_{5}$, $x_{3}$) sample respectively. In this figure, red-colored lines connect anchors and positive samples. Black-colored lines connect anchors and negative samples.

Concurrently with cross entropy the network also calculates the triplet loss in order to quantify how close intra-class embeddings and how far inter-class embeddings are. The loss function’s penalization scheme, “pushes” closer the embeddings of the same classes, and far apart those of distinct classes (Figure 3). More specifically, let $\left(x_{a},x_{p},x_{n}\right)$ be a time series triplet. A constant margin *m* is used to ensure samples with a distance greater than *m* do not contribute to the metric loss. In that sense, the triplet loss function [39] is defined as:

(3) $$L_{m}\left(x_{a},x_{p},x_{n}\right)=\frac{1}{N}\sum_{n=i}^{N}\max\left(0,D\left(x_{a},x_{p}\right)-D\left(x_{a},x_{n}\right)+m\right)$$

where $D\left(x_{i},x_{j}\right)=||f\left(x_{i}\right)-f\left(x_{j}\right){\left|\right|}_{2}$ denotes the Euclidean distance between the embeddings of $\left(x_{i},x_{j}\right)$. The classification and metric learning losses are combined through a weight λ in order to produce the total network loss $L_{t}$, where the optimal value of λ has been set empirically (details take place in the following section).

(4) $$L_{t}=L_{c}\left(x_{p},y_{p}\right)+L_{c}\left(x_{a},y_{a}\right)+L_{c}\left(x_{n},y_{n}\right)+\lambda L_{m}\left(x_{p},x_{a},x_{n}\right)$$

Consequently, with every triplet provided to the network, each of the sub-networks strives to first produce a feature embedding of it, and subsequently classify it correctly through its fully connected layers using weighted cross-entropy loss. Simultaneously, triplet losses are calculated to quantify the distances of the produced embeddings. The total loss $L_{t}$ is finally calculated in order to determine the network’s overall loss, by combining the four individual losses in Equation (4). This term is then used via back-propagation to update the weights of all three sub-networks.

## 3. Experiments

This section presents the results of a series of experiments that aim to measure if and by how much metric learning improves an LSTM network’s time series classification performance. An ablation study of the main architectural parameters has been also conducted.

### 3.1. Dataset

The Breizhcrops dataset, which we use for our experiments, has been introduced very recently [40] as a common benchmark remote sensing dataset with an emphasis on the temporal dimension. At the time of drafting this article, it has not yet been employed by researchers other than its creators [40,43]. As such, we follow their experimental setup for the sake of fairness and comparability.

**Figure 4:**
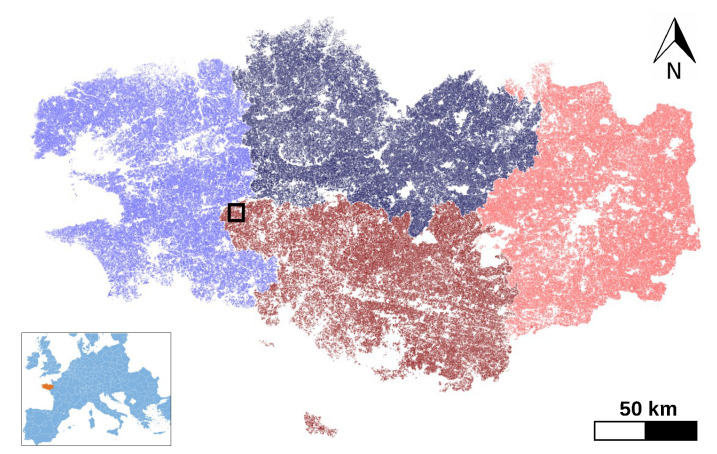
The regions of Brittany from which the dataset stems: ▬FRH01, ▬FRH02, ▬FRH03 and ▬FRH04.

The Breizhcrops dataset contains Sentinel-2 L1C data acquired regularly over the region of Brittany, France during 2017. It covers an area of approximately 27,200 km${}^{2}$ and corresponds to 7 Sentinel-2 tiles. The region is dominated by a temperate oceanic climate with an annual average temperature ranging from $5.6^{{}^{\circ}}$ in winter to $17.5^{{}^{\circ}}$ in summer and mean annual precipitation of 650 mm [43]. It possesses 13 spectral bands, labeled with also 13 agricultural classes (Table 1). The dataset consists of 765 thousand samples, each sample representing the mean-aggregated spectral response of a field parcel. Since the number of observations depends on suitable atmospheric conditions, the available sequence length per sample can vary. To standardize their temporal length, Breizhcrops samples have been set to a sequence length of 45 in the original article [40]. The dataset samples stem from four geographical regions as shown in Figure 4. Furthermore, the number of parcels of each crop type for each region is given in Table 1 together with the regional total parcel count. Although class distribution is clearly unbalanced, each region possesses all crop types all the same.

### 3.2. Settings

As far as the settings of our experiments are concerned, first of all, the Breizchcrops dataset has been employed directly in L1C level, as it has been provided [40] with no further preprocessing of any type. This decision has been made for two reasons. The first is for the sake of comparability against the results of the original publication [40] and the second is due to published reports [28,29], claiming that LSTMs achieve reasonable performance even without radiometric or geometric preprocessing.

The classification results throughout all runs, have been measured in terms of overall accuracy (OA), Cohen’s kappa score (κ), mean f1 score, mean precision and mean recall across all classes.

**Figure 5:**
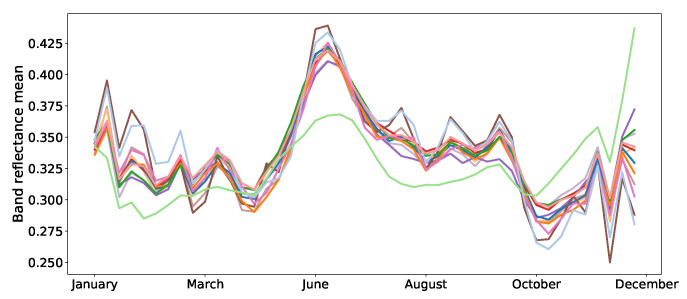
Average reflectances of the training samples: ▬rapeseed, ▬barley, ▬vegetables, ▬temp. meadows, ▬protein crops, ▬perm. meadows, ▬cereals, ▬orchards, ▬miscellaneous, ▬fallow, ▬fodder, ▬corn, ▬wheat.

As far as the data split is concerned, we created our training, validation and test sets using the same partitions as in [40]. In other words, samples of regions FRH01 and FRH02 are used for training, samples of region FRH03 are used for validation and the remaining samples of FRH04 for testing. The average reflectances of the training samples are shown in Figure 5.

Ideally, a triplet network is expected to be trained with informative triplets representing the entire spectrum of the class variances, so as to learn the finer differences among classes. More specifically, for optimal performance the network is expected to train with positive samples that are as dissimilar as possible from the anchor (i.e., hard positives), and negative samples that are as similar as possible to the anchor (i.e., hard negatives). To underline the effect of selecting positive samples too similar to the anchor and negative samples far too different from the anchor, we employ two setups for selecting our training triplets.

In setup-1, in order to better show to the network the inter-region spectral variations of the various classes, the anchor and negative samples are selected from the same region (either FRH01 or FRH02) and the accompanying positive samples from the remaining training region. In setup-2 on the other hand, triplets are naively and randomly formed, by choosing positive samples from the same class and negative samples from distinct classes using the entire training set.

**Figure 6:**
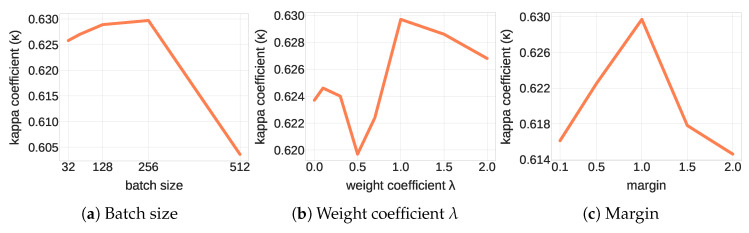
Effect on performance of various hyper-parameters of the proposed approach. (**a**) Batch size effect on performance of TripletLSTM-1. (**b**) Weight coefficient λ effect on performance of TripletLSTM-1. (**c**) Margin effect on performance of TripletLSTM-1.

The proposed TripletLSTM has been implemented with both setup-1 and setup-2 (TripletLSTM-1 and TripletLSTM-2 respectively). Its LSTM sub-networks have the same bidirectional architecture as the one used in [40]. The network is trained until its validation accuracy no longer increases. The Adam optimizer [44] with a learning rate of 0.001 has been used. We have experimented with various batch sizes, margin *m* values and λ weight factors using our validation set to determine optimal parameters. Their effect on performance is shown in Figure 6.

Both λ and the margin *m* have been found to provide optimal performance for λ=1.0 and m=1.0. The optimal batch size is set as 256, identically to the setup of [40]. Furthermore, the models have been implemented with the PyTorch framework and trained on a single K80 GPU with 25 GB of memory.

The investigated Triplet LSTM strategy is compared against multiple approaches. As a baseline we used a random forest classifier with 100 trees. Moreover, three CNN-based approaches also take place in our comparisons, namely a *Temporal CNN* (128 hidden units, filter size 7) that applies convolutions in the temporal domain, *MsResnet* (32 hidden units) equipped with residual computations, and *InceptionNet* (hidden vector dimension set to 128) known for its powerful multiscale filtering. As far as RNN-based approaches are concerned, we compare the Triplet LSTM with two variants: a vanilla LSTM of the same architecture with no metric learning, and a *StarRNN* [45] (3 layers and hidden vector dimension set to 128), known for its superior capacity in dealing with vanishing gradients.

In the context of multispectral remote sensing land cover mapping, it is common when using handcrafted features to only employ informative bands as opposed to all that are available (e.g., near infrared bands for water pollution [46]). Nevertheless, determining informative bands requires expert knowledge. Deep neural networks on the hand are commonly employed with all bands that are available [20,47,48], since it is the network itself that determines which bands will be employed for feature extraction and which not, through the weights that it learns thanks to the loss functions’ orientation. This is why the deep neural network-based approaches tested in our experiments employ all the available spectral bands.

### 3.3. Classification Results

All tested approaches have been trained and tested 5 times in order to mitigate the effect of random weight initialization. Consequently all presented scores are averages across 5 runs.

Classification results for all compared approaches are provided in Table 2. Random Forest performs the poorest as expected, since it does not take into account the sequential nature of its input, and instead treats it as a vector. CNN-based approaches on the other hand outperform random forest by 10 percentile kappa points, outlining the superiority of deep features with respect to classic machine learning techniques. The greatest performance however is achieved with RNN-based methods as they specialize in capturing temporal relations. More precisely, vanilla LSTM has a very good performance of 0.62 in terms of kappa, outperforming the recently introduced StarRNN. As far as the proposed approach is concerned, TripletLSTM-2 falls behind vanilla LSTM, thus highlighting the significance of training sample selection with metric learning. Because when care is taken to select informative triplets as in the case of TripletLSTM-1, the best performance overall with a kappa of 0.64 and an overall accuracy of 0.71 is achieved.

**Figure 7:**
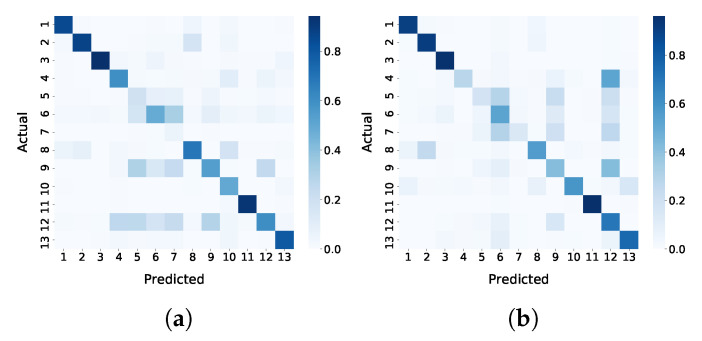
Confusion matrices for TripletLSTM-1 in terms of precision and recall. (**a**) Precision confusion matrix of TripletLSTM-1. (**b**) Recall confusion matrix of TripletLSTM-1.

**Figure 8:**
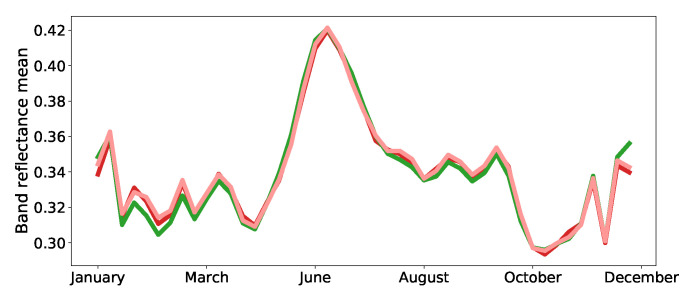
Average reflectances of the training samples belonging to ▬temp. meadows, ▬perm. meadows and ▬fodder; the most confused classes according to Figure 7.

Furthermore, based on the confusion matrices shown in Figure 7, one can observe that the classes *fodder*, *permanent meadows* and *temporary meadows* are particularly confused with each other. This is not surprising, since their profiles are highly similar (Figure 8).

**Figure 9:**
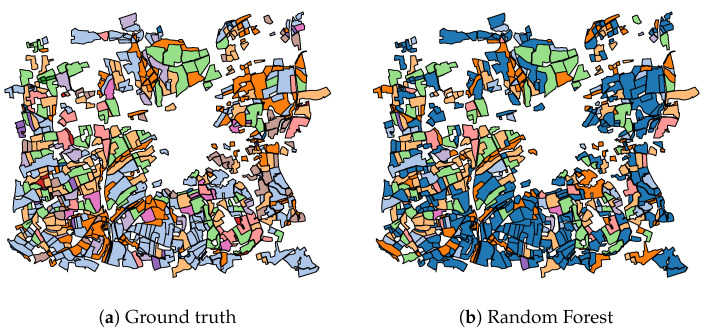
Classification map of a subset of FRH04 (Figure 4) using Random Forest: ▬rapeseed, ▬barley, ▬vegetables, ▬temp. meadows, ▬protein crops, ▬perm. meadows, ▬cereals, ▬orchards, ▬miscellaneous, ▬fallow, ▬fodder, ▬corn, ▬wheat.

**Figure 10:**
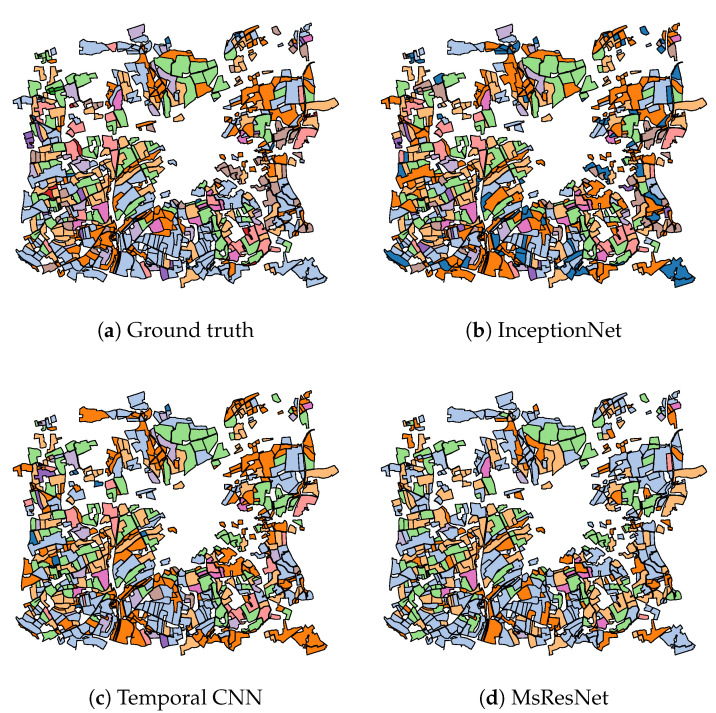
Classification maps of a subset of FRH04 (Figure 4) for CNN-based approaches: ▬rapeseed, ▬barley, ▬vegetables, ▬temp. meadows, ▬protein crops, ▬perm. meadows, ▬cereals, ▬orchards, ▬miscellaneous, ▬fallow, ▬fodder, ▬corn, ▬wheat.

**Figure 11:**
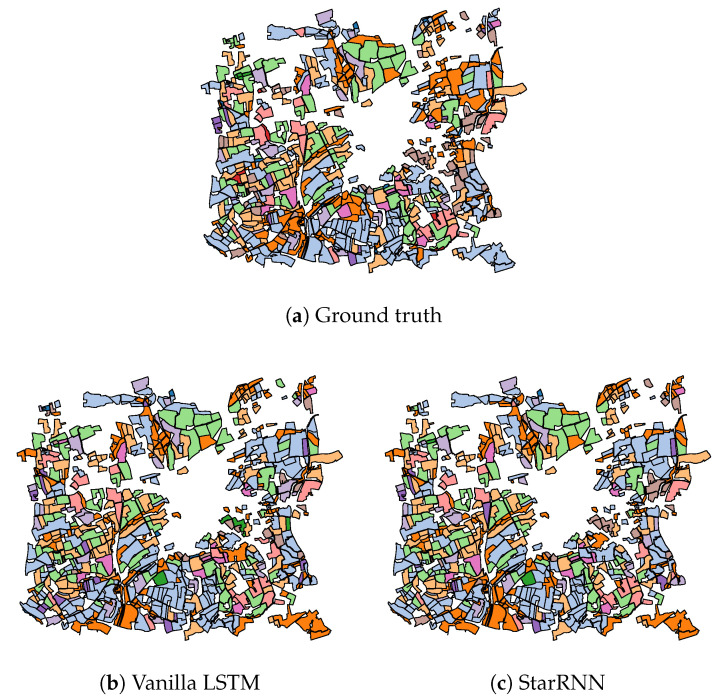

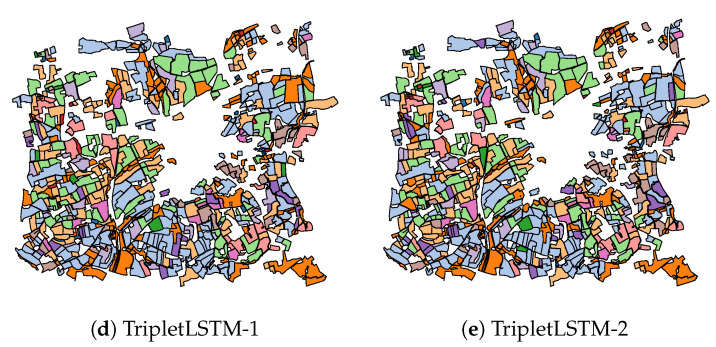
Classification maps of a subset of FRH04 (Figure 4) for RNN-based approaches: ▬rapeseed, ▬barley, ▬vegetables, ▬temp. meadows, ▬protein crops, ▬perm. meadows, ▬cereals, ▬orchards, ▬miscellaneous, ▬fallow, ▬fodder, ▬corn, ▬wheat.

In addition to the numerical results of Table 2, Figure 9, Figure 10, Figure 11 present the visual classification maps obtained via all tested approaches on a small subset of the testing region FRH04, containing roughly 750 parcels. One can immediately remark the high error level of Random Forest (Figure 9b). CNN-based approaches on the other hand (Figure 10), provide a visually apparent superior performance regarding Random Forest thanks to the superior descriptive capacity of deep features. Judging from In Figure 11, TripletLSTM-1 In (Figure 11d) appears to outperform vanilla LSTM (Figure 11b) especially with the under-represented classes such as orchards and protein crops, presumably thanks to its metric learning component.

### 3.4. Computational Complexity

The training durations of the various tested approaches are shown in Table 3, while testing durations are real-time for all approaches. They are relatively close to each other in terms of training, as well in terms of their number of network parameters, being always in the order of hundreds of thousands. The only exception is StarRNN with less than 100k parameters. LSTMs possess a particularly high number of network parameters, enabling them to capture higher level temporal relations.

## 4. Conclusions

This article addresses the topic of multi-spectral time series classification and proposes the combined use of LSTMs with metric learning, through a Triplet LSTM architecture. This is the first attempt of using LSTMs together with metric learning in the context of remote sensing data analysis.

We take advantage of weighted cross-entropy loss, to minimize classification error without allowing over-represented classes to overwhelm loss calculation. It is further combined through a weight parameter with a triplet loss to decrease the intra-class variance of the calculated feature embeddings and increase their inter-class variance.

The experiments that have been conducted with the recently introduced and challenging Breizhcrops dataset, have included several CNN and RNN variants. The proposed approach, through a careful selection of training triplets across multiple geographical regions, has outperformed its closest alternative by 2 percentile kappa points and 3 percentile overall accuracy points. Nevertheless, a performance of 0.64 in terms of kappa cannot be considered sufficient for practical purposes. Which is why we intend to further explore metric loss functions specifically crafted for multi-spectral data as well as novel triplet selection strategies.

## Figures and Tables

**Table 1:** The classes and their sizes for each region.

Class #	Crop Type	FRH01	FRH02	FRH03	FRH04
1	barley	13,046	10,733	7148	5978
2	wheat	30,368	15,005	27,189	16,993
3	corn	43,990	36,593	41,992	31,333
4	fodder	6514	4329	7639	4541
5	fallow	1521	3267	2814	4555
6	miscellaneous	17,659	12,126	21,194	15,571
7	orchards	944	350	1223	553
8	cereals	6276	3660	4516	5784
9	perm. meadows	32,650	36,512	32,534	26,117
10	protein crops	1107	461	1079	655
11	rapeseed	5593	2346	3557	3236
12	temp. meadows	52,011	39,082	52,728	38,391
13	vegetables	8538	14,266	3679	3851
	**Total**	**220,217**	**178,730**	**207,292**	**157,558**

**Table 2:** Classification performance of the various tested approaches.

Method	OA	κ	Mean f1	Mean Precision	Mean Recall
Random Forest	0.545	0.441	0.295	0.339	0.312
Temporal CNN	0.624	0.546	0.440	0.554	0.440
MsResNet	0.632	0.565	0.502	0.658	0.491
InceptionNet	0.632	0.570	0.535	0.591	0.533
StarRNN	0.664	0.596	0.529	0.582	0.530
Vanilla LSTM [40]	0.680	0.620	0.590	0.630	0.580
TripletLSTM-1	**0.711**	**0.641**	**0.631**	**0.642**	**0.620**
TripletLSTM-2	0.678	0.619	0.588	0.590	0.604

**Table 3:** Training durations in minutes and network complexity.

Method	Training Duration	Number of Network Parameters
Random Forest	39	-
Temporal CNN	36	787,085
MsResNet	30	537,325
InceptionNet	37	215,053
StarRNN	39	72,103
Vanilla LSTM [40]	33	942,376
TripletLSTM-1	39	942,375
TripletLSTM-2	37	942,375

## References

[1] Belgiu M., Csillik O. (2018). Sentinel-2 cropland mapping using pixel-based and object-based time-weighted dynamic time warping analysis. Remote Sens. Environ..

[2] Gomez-Chova L., Tuia D., Moser G., Camps-Valls G. (2015). Multimodal Classification of Remote Sensing Images: A Review and Future Directions. Proc. IEEE.

[3] Defourny P., Bontemps S., Bellemans N., Cara C., Dedieu G., Guzzonato E., Hagolle O., Inglada J., Nicola L., Rabaute T. (2019). Near real-time agriculture monitoring at national scale at parcel resolution: Performance assessment of the Sen2-Agri automated system in various cropping systems around the world. Remote Sens. Environ..

[4] Xiong J., Thenkabail P.S., Gumma M.K., Teluguntla P., Poehnelt J., Congalton R.G., Yadav K., Thau D. (2017). Automated cropland mapping of continental Africa using Google EarthEngine cloud computing. Remote Sens. Environ..

[5] Yan L., Roy D.P. (2015). Improved time series land cover classification by missing-observation-adaptive nonlinear dimensionality reduction. Remote Sens. Environ..

[6] Solano-Correa Y.T., Bovolo F., Bruzzone L. A Semi-supervised Crop-type Classification Based on Sentinel-2 NDVI Satellite Image Time Series and Phenological Parameters. Proceedings of the IEEE International Geoscience and Remote Sensing Symposium.

[7] Petitjean F., Inglada J., Gancarski P. (2012). Satellite image time series analysis under time warping. IEEE Trans. Geosci. Remote Sens..

[8] Petitjean F., Weber J. (2014). Efficient Satellite Image Time Series Analysis Under Time Warping. IEEE Trans. Geosci. Remote Sens..

[9] Csillik O., Belgiu M., Asner G.P., Kelly M. (2019). Object-Based Time-Constrained Dynamic Time Warping Classification of Crops Using Sentinel-2. Remote Sens..

[10] Furby S., Caccetta P., Wu X., Chia J. Continental scale land cover change monitoring in Australia using Landsat imagery. Proceedings of the International Earth Conference: Studying, Modeling and Sense Making of Planet Earth.

[11] Loveland T.R., Reed B.C., Brown J.F., Ohlen D.O., Zhu Z., Yang L., Merchant J.W. (2000). Development of a global land cover characteristics database and IGBP DISCover from 1 km AVHRR data. Int. J. Remote Sens..

[12] Zhang Z., Tang P., Corpetti T. Satellite image time series clustering via affinity propagation. Proceedings of the IEEE International Geoscience and Remote Sensing Symposium.

[13] Gebhardt S., Wehrmann T., Muñoz Ruiz M.A., Maeda P., Bishop J., Schramm M., Kopeinig R., Cartus O., Kellndorfer J., Ressl R. (2014). MAD-MEX: Automatic wall-to-wall land cover monitoring for the Mexican REDD-MRV program using all Landsat data. Remote Sens..

[14] Clark M.L., Aide T.M., Rinera G. (2012). Land change for all municipalities in Latin America and the Caribbean assessed from 250-m MODIS imagery (2001–2010). Remote Sens. Environ..

[15] Inglada J., Vincent A., Arias M., Tardy B., Morin D., Rodes I. (2017). Operational high resolution land cover map production at the country scale using satellite image time series. Remote Sens..

[16] Pelletier C., Valero S., Inglada J., Champion N., Dedieu G. (2016). Assessing the robustness of Random Forests to map land cover with high resolution satellite image time series over large areas. Remote Sens. Environ..

[17] Gomez C., White J.C., Wulde M.A. (2016). Optical remotely sensed time series data for land cover classification: A review. ISPRS J. Photogramm. Remote Sens..

[18] Hinton G.E., Salakhutdinov R.R. (2006). Reducing the dimensionality of data with neural networks. Science.

[19] Krizhevsky A., Sutskever I., Hinton G.E. ImageNet classification with deep convolutional neural networks. Proceedings of the International Conference on Neural Information Processing Systems.

[20] Ma L., Liu Y., Zhang X., Ye Y., Yin G., Johnson B.A. (2019). Deep learning in remote sensing applications: A meta-analysis and review. ISPRS J. Photogramm. Remote Sens..

[21] Hochreiter S., Schmidhuber J. (1997). Long short-term memory. Neural Comput..

[22] Sun Y., Luo J., Wu T., Yang Y., Liu H., Dong W., Gao L., Hu X. Geo-parcel based Crops Classification with Sentinel-1 Time Series Data via Recurrent Reural Network. Proceedings of the IEEE International Conference on Agro-Geoinformatics.

[23] Sun Z., Di L., Fang H. (2018). Using Long Short-Term Memory Recurrent Neural Network in land cover classification on Land-sat and Cropland data layer time series. Int. J. Remote Sens..

[24] Zhong L., Hu L., Zhou H. (2019). Deep learning based multi-temporal crop classification. Remote Sens. Environ..

[25] Avolio C., Tricomi A., Mammone C., Zavagli M., Costantini M. A deep learning architecture for heterogeneous and irregularly sampled remote sensing time series. Proceedings of the IEEE International Geoscience and Remote Sensing Symposium.

[26] Garnot V.S.F., Landrieu L., Giordano S., Chehata N. Time-Space Tradeoff in Deep Learning Models for Crop Classification on Satellite Multi-Spectral Image Time Series. Proceedings of the IEEE International Geoscience and Remote Sensing Symposium.

[27] Zhou Y., Luo J., Fen L., Zhou X. (2019). DCN-Based Spatial Features for Improving Parcel-Based Crop Classification Using High-Resolution Optical Images and Multi-Temporal SAR Data. Remote Sens..

[28] Rußwurm M., Körner M. Temporal Vegetation Modelling Using Long Short-Term Memory Networks for Crop Identification from Medium-Resolution Multi-spectral Satellite Images. Proceedings of the Computer Vision and Pattern Recognition Workshops.

[29] Rußwurm M., Körner M. (2018). Multi-Temporal Land Cover Classification with Sequential Recurrent Encoders. ISPRS Int. J. Geo Inf..

[30] Pelletier C., Webb G.I., Petitjean F. Deep Learning for the Classification of Sentinel-2 Image Time Series. Proceedings of the IEEE International Geoscience and Remote Sensing Symposium.

[31] Xing E.P., Ng A.Y., Jordan M.I., Russel S. Distance Metric Learning, with Application to Clustering with Side-Information. Proceedings of the International Conference on Neural Information Processing.

[32] Tian Z., Zhang Z., Mei S., Jiang R., Wan S., Du Q. Discriminative CNN via Metric Learning for Hyperspectral Classification. Proceedings of the IEEE International Geoscience and Remote Sensing Symposium.

[33] Liu Y., Huang C. (2018). Scene classification via triplet networks. IEEE J. Sel. Top. Appl. Earth Obs. Remote Sens..

[34] Cheng G., Yang C., Yao X., Guo L., Han J. (2018). When Deep Learning Meets Metric Learning: Remote Sensing Image Scene Classification via Learning Discriminative CNNs. IEEE Trans. Geosci. Remote Sens..

[35] Liu X.N., Zhou Y., Zhao J.Q., Yao R., Liu B., Zheng Y. (2019). Siamese Convolutional Neural Networks for Remote Sensing Scene Classification. IEEE Trans. Geosci. Remote Sens..

[36] Bromley J., Guyon I., LeCun Y., Säckinger E., Shah R. Signature Verification using a “Siamese” Time Delay Neural Network. Proceedings of the Conference on Neural Information Processing Systems.

[37] Zhan Y., Fu K., Yan M., Sun X., Wang H., Qiu X. (2017). Change Detection Based on Deep Siamese Convolutional Network for Optical Aerial Images. IEEE Trans. Geosci. Remote Sens..

[38] Chopra S., Hadsell R., LeCun Y. Learning a similarity metric discriminatively, with application to face verification. Proceedings of the IEEE Conference on Computer Vision and Pattern Recognition.

[39] Hoffer E., Ailon N. Deep Metric Learning Using Triplet Network. Proceedings of the International Workshop on Similarity-Based Pattern Recognition.

[40] Rußwurm M., Lefèvre S., Körner M. BreizhCrops: A Time Series Dataset for Crop Type Identification. Proceedings of the Time Series Workshop of the 36th International Conference on Machine Learning.

[41] Bosilj P., Aptoula E., Duckett T., Cielniak G. (2020). Transfer learning between crop types for semantic segmentation of crops versus weeds in precision agriculture. J. Field Robot..

[42] Eigen D., Fergus R. Predicting depth, surface normals and semantic labels with a common multi-scale convolutional architecture. Proceedings of the IEEE International Conference on Computer Vision.

[43] Rußwurm M., Pelletier C., Zollner M., Lefèvre S., Körner M. (2020). BreizhCrops: A Time Series Dataset for Crop Type Mapping. arXiv.

[44] Kingma D., Ba J. Adam: A method for stochastic optimization. Proceedings of the International Conference on Learning Representation.

[45] Turkoglu M.O., D’Aronco S., Wegner J.D., Schindler K. (2019). Gating Revisited: Deep Multi-layer RNNs That Can BeTrained. arXiv.

[46] Pahlevan N., Smith B., Schalles J., Binding C., Cao Z., Mae R., Alikas K., Kangrof K., Gurling D., Hà N. (2020). Seamless retrievals of chlorophyll-a from Sentinel-2 (MSI) and Sentinel-3 (OLCI) in inland and coastal waters: A machine-learning approach. Remote Sens. Environ..

[47] Ienco D., Gaetano R., Dupaquier C., Maurel P. (2017). Land Cover Classification via Multitemporal Spatial Data by Deep Recurrent Neural Networks. IEEE Trans. Geosci. Remote Sens..

[48] Kussul N., Lavreniuk M., Skakun S., Shelestov A. (2017). Deep learning classification of land cover and crop types using remote sensing data. IEEE Trans. Geosci. Remote Sens..

